# The formation of impact coesite

**DOI:** 10.1038/s41598-021-95432-6

**Published:** 2021-08-06

**Authors:** F. Campanale, E. Mugnaioli, M. Gemmi, L. Folco

**Affiliations:** 1 Dipartimento di Scienze della Terra, Università d Pisa, Via S. Maria 53, 56126 Pisa, Italy; 2 Center for Nanotechnology Innovation@NEST, Istituto Italiano di Tecnologia (IIT), Piazza San Silvestro 12, 56127 Pisa, Italy; 3 CISUP, Centro per l’Integrazione della Strumentazione dell’Università di Pisa, Lungarno Pacinotti 43, 56126 Pisa, Italy

**Keywords:** Planetary science, Mineralogy

## Abstract

Coesite in impact rocks is traditionally considered a retrograde product formed during pressure release by the crystallisation of an amorphous phase (either silica melt or diaplectic glass). Recently, the detailed microscopic and crystallographic study of impact ejecta from Kamil crater and the Australasian tektite strewn field pointed in turn to a different coesite formation pathway, through subsolidus quartz-to-coesite transformation. We report here further evidence documenting the formation of coesite directly from quartz. In Kamil ejecta we found sub-micrometric single-coesite-crystals that represent the first crystallization seeds of coesite. Coesite in Australasian samples show instead well-developed subeuhedral crystals, growing at the expenses of hosting quartz and postdating PDF deformation. Coesite (010) plane is most often parallel to quartz {10–11} plane family, supporting the formation of coesite through a topotactic transformation. Such reaction is facilitated by the presence of pre-existing and shock-induced discontinuities in the target. Shock wave reverberations can provide pressure and time conditions for coesite nucleation and growth. Because discontinuities occur in both porous and non-porous rocks and the coesite formation mechanism appears similar for small and large impacts, we infer that the proposed subsolidus transformation model is valid for all types of quartz-bearing target rocks.

## Introduction

Coesite is one of the most common and reliable indicator of impact cratering in quartz-bearing target rocks. Its formation conditions have been a debated issue since its discovery in nature by Chao et al.^1^. Three models of coesite formation have been proposed: (1) crystallisation during decompression from silica melt with short-range order and silicon in fourfold coordination^2–7^ or (2) crystallisation in solid-state condition from diaplectic silica glass^8^; (3) direct solid-state quartz-to-coesite transformation in thermodynamically non-equilibrium conditions^9–11^. The models (1) and (2) are essentially based on the study of non-porous target rocks and single quartz crystals, while model (3) derives from the study of porous sandstones.

In this paper we present new FEG-SEM, TEM and 3D ED coesite microstructures from shocked sandstones from the Kamil crater, a 45 m diameter crater located in the East Uweinat Desert, Egypt^12^, and from microscopic ejecta from the Australasian tektite/microtektite strewn field, which extends for over 15% of the Earth's surface^13^. We will show that these new features document the direct formation of coesite from shocked quartz under subsolidus conditions during the shock compression and emphasize the important role of structural discontinuities in the target for its nucleation. Such a model poses new constraints on the peak shock pressures experienced by coesite bearing impact rocks and appear independent from the type of the target rock, e.g. porous versus crystalline. We also postulate that the final quartz-to-coesite volume ratio is strongly related with the pressure pulse duration, rather than the maximum pressure peak associated with the shock event.

## Results

### Kamil crater shocked arenite

Sample L23 is a pale, medium-grained quartz arenite ejecta showing an extraordinary variety of shock metamorphic features (e.g. shocked zircon^14^ and high-pressure silica structures, including fractures, planar deformation features [PDFs], microcrystalline coesite, and silica glass^12^). In particular, the studied section hosts a great abundance of coesite, mainly concentrated in the so-called symplectic regions—i.e. intergranular veins and pockets of silica glass in between quartz grains^10^. Note that in this paper the term ‘silica glass’ is only used to indicate silica material which is structurally amorphous, with no genetic or further structural implications.

**Figure 1:**
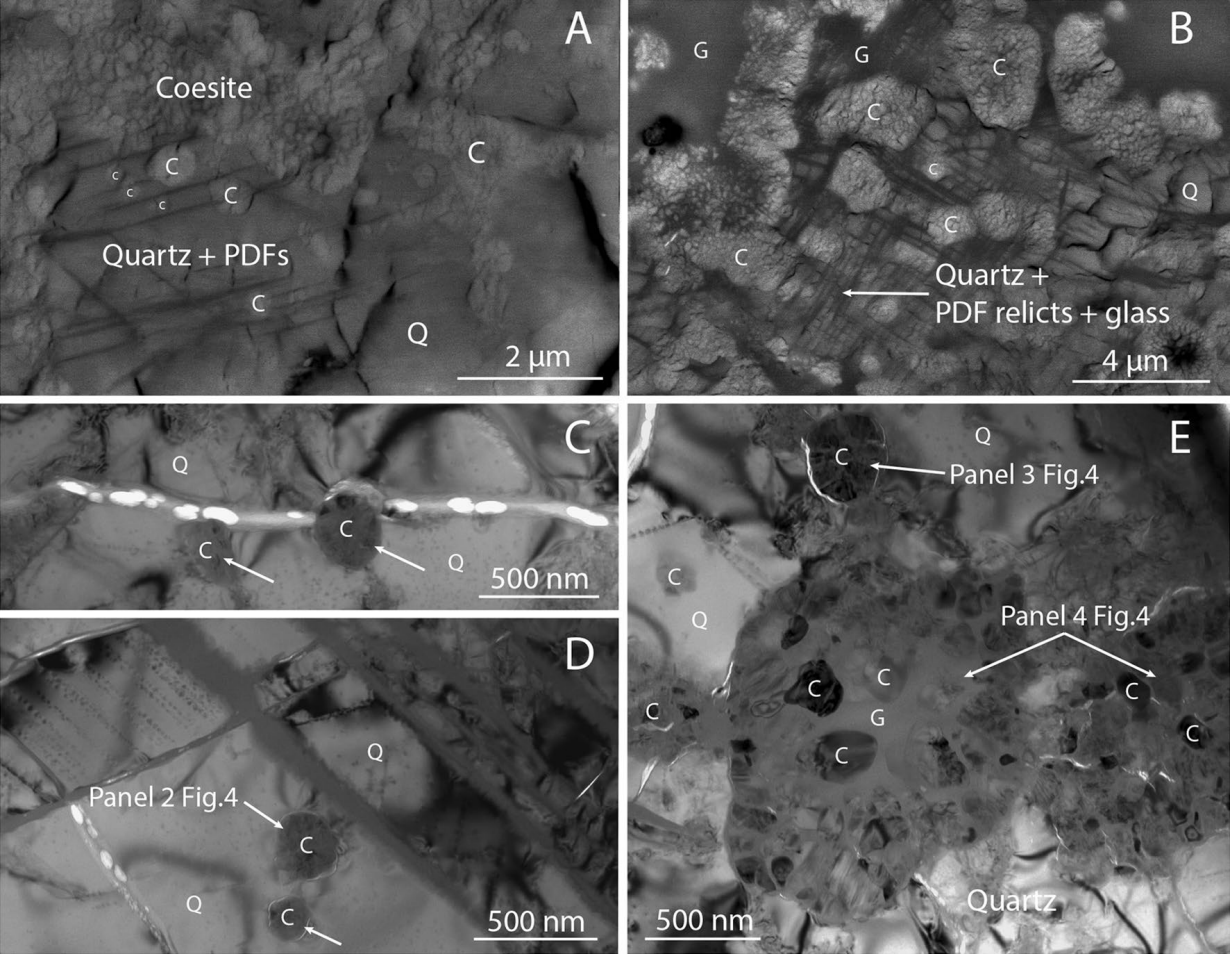
Coesite-quartz intergrowth in shocked quartz arenite from Kamil crater. (**A**) BSE image showing at least two sets of PDFs in quartz, overgrown by spheroidal coesite grains and large coesite aggregates. (**B**) BSE image showing coesite aggregates, subrounded in shape, embedded in silica glass and deformed PDF-bearing quartz relicts. (**C**) Bright field TEM image showing the direct contact quartz–coesite: two single-crystal coesite (indicated by arrows) within quartz and bordered by a fracture. The 3D ED volume of the single-crystal coesite on the right is shown in Fig. 3. (**D**) TEM image showing other spheroidal coesite single-crystals in quartz (arrows), apparently not in contact with PDFs. (**E**) TEM image showing in the upper part a slightly larger spheroidal coesite looking mottled or scaly, due to the presence of partially misaligned coesite fragments. At the centre of the image two larger rounded coesite aggregates with different amount of silica glass. Some features are labelled to be compared with the related sketches in Fig. 4. C = coesite, Q = quartz, G = glass.

In this study, we focused on a region 120 × 60 µm in size (see Supplementary material) which is close to an important vein filled with silica glass that crosses one-third of the thin section. This region consists of shocked PDF-bearing quartz grains with size of about 5 to 20 µm, separated by intergranular veins and pockets of silica glass. Back-scattered electron (BSE) images show that coesite forms rounded micrometre-sized aggregates of bright (high BSE contrast) nano-crystals (Fig. 1A). Aggregates appear scaly due to the intercalation of thin films of a darker (low BSE contrast) groundmass, later identified as silica glass by TEM. Coesite aggregates can be found entirely within quartz crystals (Fig. 1A) or surrounded by a matrix of silica glass and deformed PDF-bearing quartz relicts (Fig. 1B). Coesite appears particularly abundant along fractures and veins of silica glass. Some aggregates show a darker BSE signal at the core, possibly due to reduced density or crystallinity (Fig. 1B). Quartz grains show PDFs, with at least two different sets intercepting each other (Fig. 1A) and  with a variable spacing among the lamellae ranging from few nm up to ~ 0.5 µm. In the same cases, coesite grows among the sets of PDFs, and when they get in contact, the PDFs look occasionally interspersed by coesite grains (see red lines in Fig. 1A,B).

TEM analysis of FIB lamellae confirms the presence of coesite, entirely hosted within single-crystal quartz grains. Quartz always displays the same crystallographic orientation inside a single FIB lamella, and it is therefore reasonable to assume that it was a single crystalline grain before the shock event. Coesite forms micro- to nano-metric grains with an overall spheroidal outline. The smaller spheroidal coesite grains are single crystals, generally 0.1 µm to 0.3 µm in size and circular in two dimensions (Fig. 1C,D). They show a uniform or slightly mottled contrast and deliver a single-crystal ED pattern. No glass phase is present between their rim and the hosting quartz. Larger spheroidal coesites (about 0.3 to 1 μm) appear mottled or scaly, with the aspect of a jigsaw of smaller and partially misaligned fragmented grains (Fig. 1E up). Diffraction reflections from these coesites are smeared in small arcs, indicating a small misorientation of the grains, which still preserve the memory of the original single crystal (Fig. 3 top-left). Coesite also appears as rounded aggregates with size up to several micrometers (Fig. 1E down), consisting of well-separated and randomly aligned coesite crystals in silica glass (the low BSE contrast groundmass mentioned above). These coesite crystals look anhedral and irregular in shape and some of these show planar contrast features that are, in all likelihood, the typical polysynthetic twinning of impact coesite along (010) planes^15^.

3D ED reveals that the smallest spheroidal coesite single-crystals are oriented so that coesite (010) is parallel to quartz {10–11} or to quartz {− 1011} (Fig. 3), coherently with what observed by Campanale et al.^11^ in the Australasian ejecta. We also attempted a dynamical refinement of quartz data for determining its absolute structure in space group *P*3_1_21 or *P*3_2_21, with the method proposed by Brázda et al.^16^ However, we did not attain a convincing result, probably due to the relatively larger thickness of FIB cuts compared to isolated nano-crystals. Therefore, we could not solve the ambiguity existing between {10–11} and {− 1011} quartz planes, which are geometrically not distinguishable but structurally different (Fig. 3).

### Australasian tektite/microtektite strewn field

The two Australasian coesite-bearing quartz ejecta studied in this work are silica shocked particles, < 300 µm^2^ in size, bearing coesite and PDFs as major shock metamorphic features (see Supplementary material). A detailed FEG-SEM and Raman description of these particles is reported in Campanale et al.^11^.

**Figure 2:**
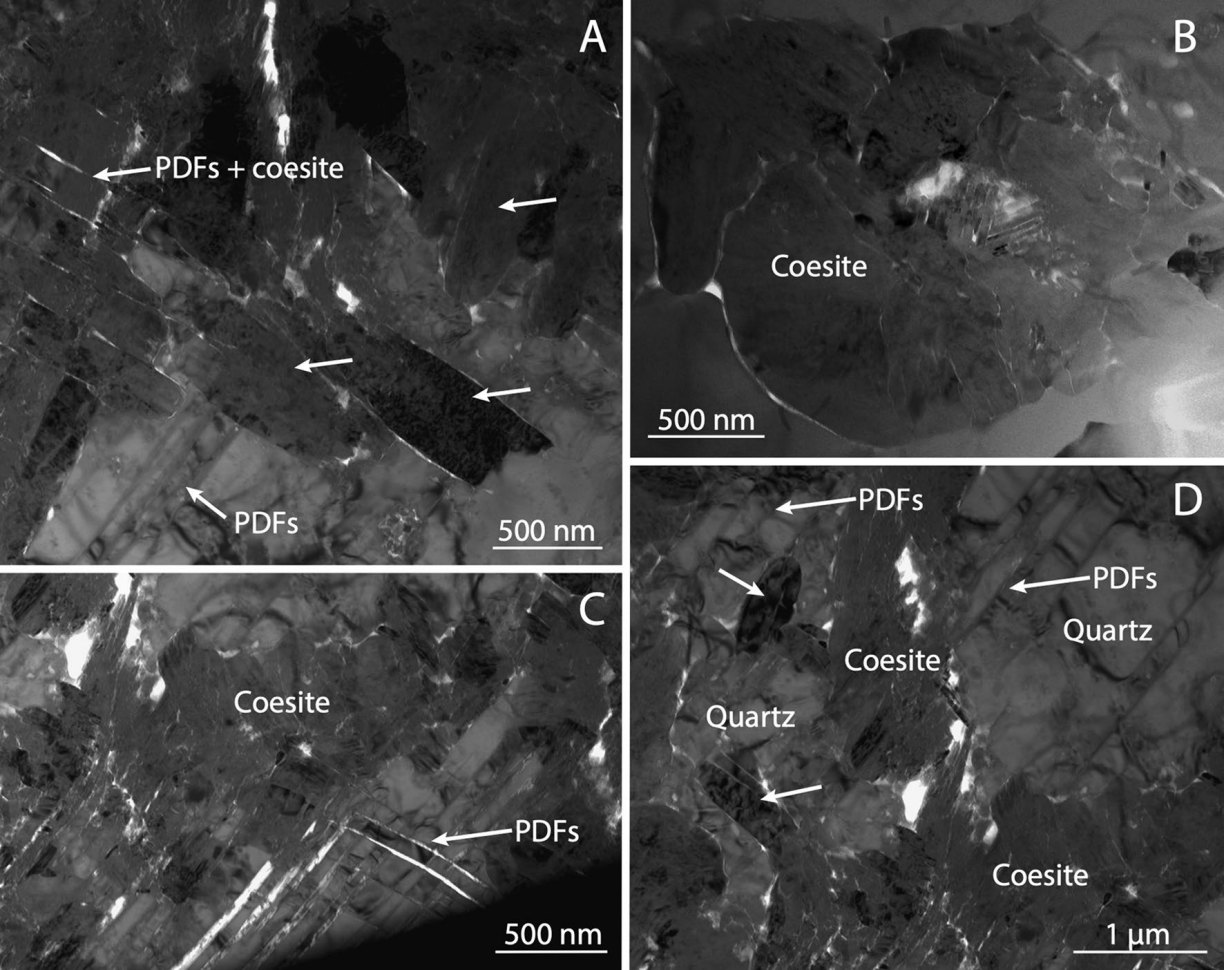
Coesite-quartz intergrowth in silica impact ejecta from the Australasian tektite strewn field. (**A**) Bright field TEM image of subeuhedral and elongated coesite crystals (see arrows) growing in direct contact with quartz. On the left side, there are partially erased or resorbed sets of PDFs within coesite area. Other PDFs appear on the bottom-left and stop at the coesite border. (**B**) TEM image of a rounded coesite aggregate of crystals ~ 1 µm in size and in direct contact with quartz. The 3D ED volume of the labelled coesite is shown in Fig. 3  top-right. (**C**,**D**) TEM image showing the intergrowth between coesite and quartz. The PDFs extend from quartz grains to the coesite grains, where they look progressively erased or resorbed.

The FIB lamellae studied for this paper consist of variable amounts of coesite and quartz (Fig. 2). Quartz shows always the same orientation inside the whole FIB lamella and is usually intersected by two sets of PDFs (Fig. 2A,C). PDFs have spacing from 50 to 700 nm and thickness of ~ 15–20 nm. Their rims can be close or partially open. Silica glass is essentially absent in the lamellae, or at most concentrated in very thin layers along the close PDFs.

**Figure 3:**
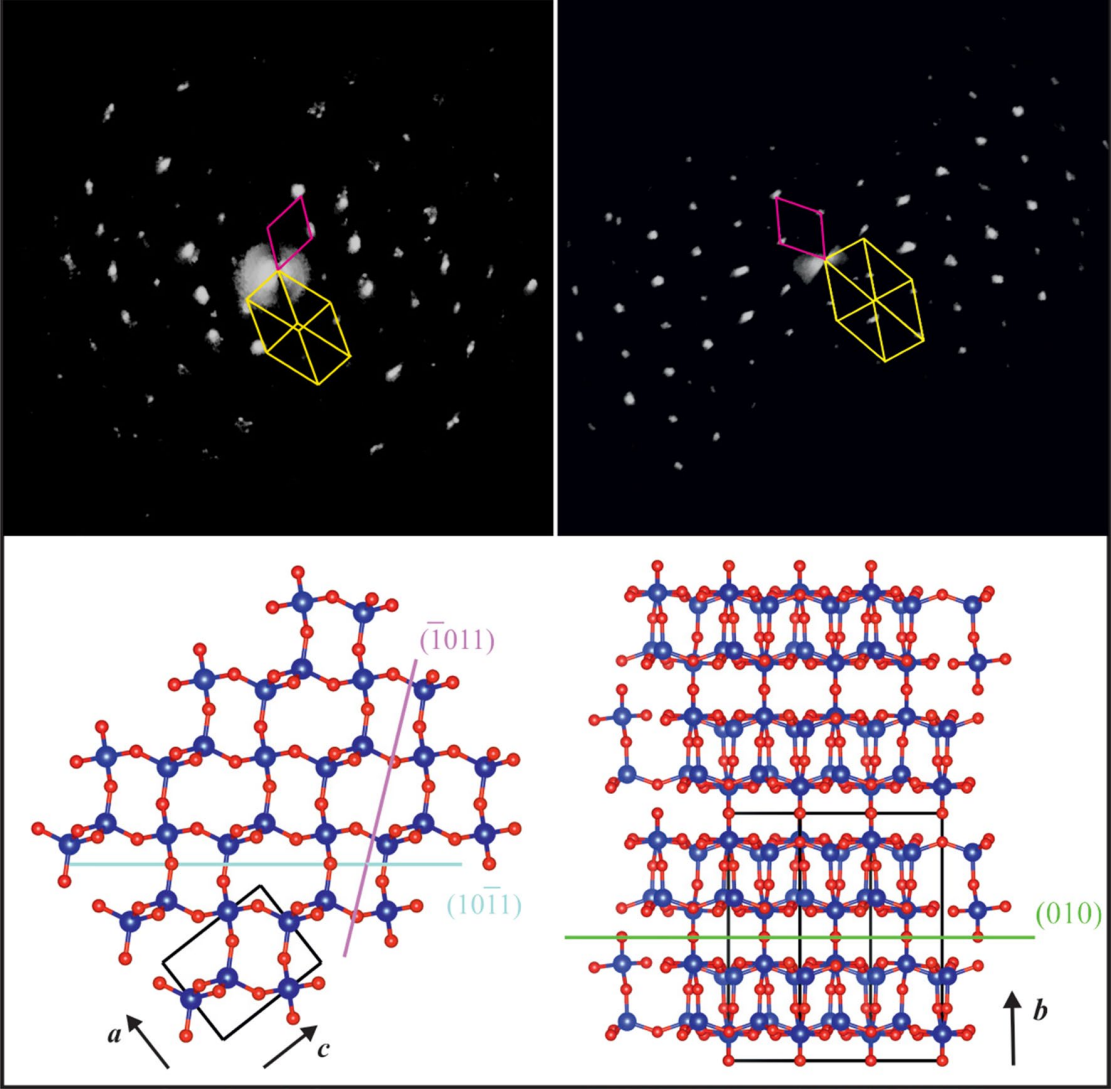
Electron diffraction data and comparison between the crystal structures of quartz and coesite. The top-left and top-right images show a reconstructed 3D ED volume of coesite from Kamil crater (see Fig. 1C) and from the Australasian strewn field (see Fig. 2B), respectively, with superimposed the related unit cell (purple). For comparison, the unit cell of the neighbouring quartz is also shown in yellow. It is evident that the (010) of coesite is almost parallel to the {10–11} or {− 1011} of quartz. In the diffraction of coesite from Kamil, the reflections are dispersed in small arcs indicating misorientation of the grains. The bottom-left image is the *P*3_1_21 quartz structure viewed along *b*, with (− 1011) and (10–11) planes drawn in blue and purple, respectively. The bottom-right image is the coesite structure viewed along [201], with (010) plane drawn in green.

Compared to the samples studied by Campanale et al.^11^, the FIB lamellae analysed here show coesite single crystals with larger size, up to ~ 1.5 μm. Typically, coesites have elongated subeuhedral habit (Fig. 2A), but rounded aggregates are also rather common (Fig. 2B). Most crystals show polysynthetic twinning along (010) planes when properly oriented (Fig. 2C). Coesite is clearly hosted inside quartz, with no glass phase at the rim. In certain cases, coesite elongation follows quartz PDF orientation, while in other cases PDFs fade or are cut by coesite grains.

3D ED data were acquired from seven, relatively large coesite crystals and from the surrounding quartz area. The geometrical superposition of the related reciprocal space reconstructions revealed that for five coesite grains, coesite plane (010) was parallel to quartz plane families {10–11} or {− 1011}, as already reported by Campanale et al.^11^. The last two coesite belonged to a rounded polycrystalline aggregate (Fig. 2B). For one of these crystals, (010) plane was again parallel to embedding quartz {10–11} or {− 1011}, while for the other (010) plane was parallel to quartz {1–321} or {− 13–21}.

## Discussion

The samples from the Australasian strewn field and Kamil crater show evidence of direct contact between quartz and coesite, with individual crystals of coesite that are in some cases entirely located within shocked quartz grains. However, the micro-petrographic setting of coesite appears different in the two samples. In the Australasian micro-ejecta, coesite occurs as subeuhedral crystals up to 1.5 µm in size, growing either within quartz grains, possibly among sets of PDFs (Fig. 2). In the reported occurrence from Kamil crater, coesite appears with a spheroidal shape either fully within quartz, or at PDFs and fractures, or at grain boundaries (Fig. 1). The smallest spheroidal coesite are single crystals, while the larger appear partially fragmented in several coesite grains, which get progressively more disoriented and more intercalated by amorphous silica up to forming polycrystalline coesite aggregates. In any case, coesite appears to interrupt planar discontinuities and this may indicate that coesite formation postdate PDFs and fractures.

These features appear difficult to explain through models that predict crystallisation of coesite during decompression, either from a silica melt with short-range order in fourfold coordination^2^ or from a solid-state diaplectic silica glass^8^. Conversely, they strongly suggest a direct quartz-to-coesite transformation in subsolidus conditions^9–11^, with coesite nucleating and growing at the expenses of pre-existing quartz, and after PDF formation, followed by melting and resorption. The petrographic evidence for the direct quartz-to-coesite transformation is supported by electron diffraction data, which reveals that, most often, the coesite plane (010) is parallel to quartz {10–11} or {− 1011} plane families.

This evidence may be consistent with coesite crystals that transform directly from quartz through a topotactic transformation. In this regard, Campanale et al.^11^ proposed a martensitic-like subsolidus mechanism (Fig. 3) studying the impact ejecta from the Australasian tektite strewn field consistently with the typical polysynthetic planar twinning of impact coesite. Alternatively, an epitactic precipitation of coesite on a crystalline quartz substrate could also be assumed. We would anyway exclude this second hypothesis, because there is no evidence of intervening fluid, amorphous, or third phase intermediate in neither the Australasian samples (Fig. 2) nor in correspondence of the smallest and more coherent coesite single-crystals in the Kamil samples (Fig. 1C,D). Moreover, coesite appears to grow at the expense of quartz, as expected in a typical topotactic reaction. Conversely, large coesite aggregates do contain a large fraction of amorphous silica, together with isolated crystalline coesite grains (Fig. 1E down) that, when found alone, may be interpreted as crystallised from the amorphous substrate. Still, such aggregates show corrosion microtexture, individual grains have random orientations, and in general they are not in contact with the surrounding quartz, and therefore they do not show any epitactic relation.

There is also the possibility that, even at the same crater, the coesite formed through two or more different mechanisms, as proposed by other authors^8,9^. Kieffer et al.^9^, for instance, carried out a detailed TEM investigation of the shocked Coconino sandstone from Meteor crater, finding evidence of both direct quartz-to-coesite transition and crystallization from silica melt. The authors supported the former model by observing the direct contact between quartz and rounded subhedral coesite with a preferred orientation, similarly to what reported here for Kamil crater. The latter model was instead supported by the finding of polygonal and equidimensional coesite grains with triple junction at the grain boundary (120° angles between them). Yet, in the samples studied in this work we did not find any evidence supporting the second coesite formation model, neither at Kamil crater nor at the Australasian tektite strewn field.

Concerning the observed crystallographic relationship between quartz and coesite, it is worth mentioning that {10–11} is a recurrent orientation for planar fractures, cleavage fractures and occasionally PDFs in shocked quartz. This suggests a genetic relation of such deformation features and coesite. Nevertheless, there is a crystallographic ambiguity between {10–11} and {− 1011} plane families, which are geometrically indistinguishable but correspond to different structural planes (Fig. 3). The analysis is further complicated because quartz has a chiral structure and can crystallise in both space groups *P*3_1_21 and *P*3_2_21. To discriminate {10–11} or {− 1011} plane families one should rely on dynamical scattering, which imply extremely high-quality electron diffraction data on very thin samples. In any case, the qualitative analysis of quartz crystal structure suggests that {10–11} is the weaker plane where a relative shift can more likely occur, assuming a quartz model in *P*3_1_21^17^. We therefore postulate that the smallest spheroidal coesite single-crystal observed in the Kamil crater shocked quartz-arenite represent the initial stage of coesite nucleation during the propagation of the compressional shock wave.

**Figure 4:**
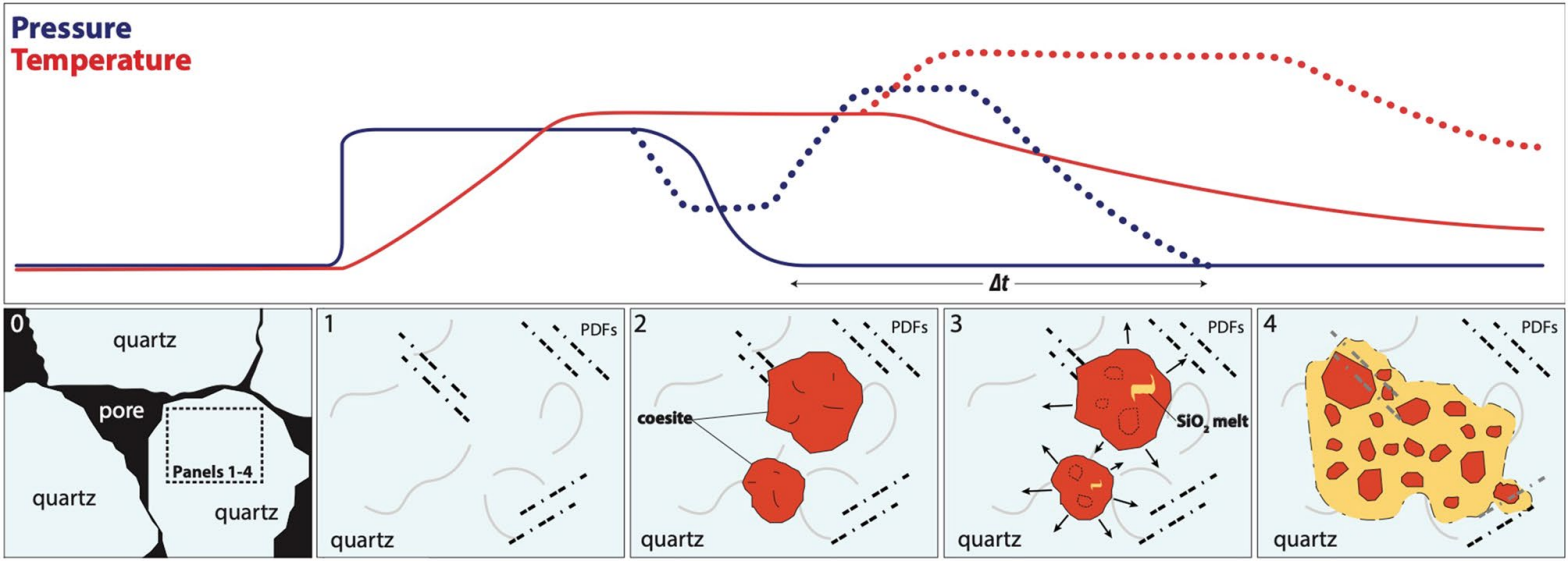
Sketch model of coesite formation and evolution in shocked quartz arenite from Kamil crater (Egypt). Solids lines in the chart above refer to the average pressure and temperature (blue and red solid lines, respectively) experienced by the target rock due to the primary shock wave. Dotted lines refer to the local pressure and temperature at discontinuities (see below). The time scale is not linear. Panel 0: sandstone porous target rock prior to the impact event. Panel 1: the extremely fast passage of the shock wave during the loading stage results in the formation of a wide range of shock features in the target quartz grains, such as fractures (grey lines) and PDFs (dashed lines). Panel 2: during compression, the average pressure remains constant for milliseconds up to seconds for very large impact events. Locally, however, the peak shock pressure experiences oscillations (see blue dotted line) due to shock wave reverberations at structural discontinuities, such as pores, grain boundaries, fractures, PDFs and crystal defects. This way pressure remains here above the quartz stability field for a sufficiently longer time for coesite nucleation and single-crystal growth. Panel 3: the decompression causes expansion, breaking of the spheroidal coesite single-crystal resulting in fragments of single crystal formation and local incipient melting within. At this stage, the fragments of an original single crystal have still the same crystallographic orientation. Note that ∆t represents the increase in time under which the local volume experience high pressure due to shock wave reverberation. Panel 4: Decompression melting proceeds, resulting in the coesite aggregates formation due to the partial fusion of coesite fragments and surrounding quartz. Coesite fragments ‘float’ in the melt, losing their original crystallographic iso-orientation. The faster the temperature decreases, the more coesite remains. At this stage, more coesite domains may merge each other forming interconnected veins and pockets filled by silica glass.

A possible mechanism for the nucleation and evolution of coesite at Kamil crater is illustrated in Fig. 4. The propagation of the shock wave in the quartz-arenite leads to the formation of new fractures, PDFs, and other defects in addition to the pre-existing discontinuities in the porous target rock (panel 1). At this stage, a peak shock pressure estimation of 20–25 GPa (on single quartz crystal)^2^ or ~ 15 GPa (on 25–30 vol% porous quartz-bearing rocks)^10,12,19^ was probably reached, considering the recurrent {10–13}, {10–12}, {10–14}, {10–11} and {11–22} PDF orientations found at Kamil^12^ and the amount of high-pressure silica polymorphs and glass^10^. Where the pressure pulse was sufficient, coesite formation took place at metastable conditions as tiny spheroidal grains (< 500 nm) within shocked quartz, through a direct subsolidus transformation (panel 2 of Fig. 4; Fig. 1C,D). This nucleation was probably favoured by localized shock-wave reverberation^10,18^ at quartz discontinuities, such us fractures, crystal defects and boundaries or other pre-existing heterogeneities in the porous target rock. This process induces localized pressure drops and subsequent pressure and temperature amplifications in the adjacent quartz grains, so that pressure and temperatures can locally remain in the coesite stability field the time necessary for coesite nucleation. The presence of initial porosity may increase the internal energy of the shock compression, allowing the system to reach the temperature required to overcome the high activation energies for mineral phase transitions^20^. Moreover, we found evidence suggesting that PDFs, as well as brittle deformation features, occurred before coesite nucleation and growth. Thus, such features might as well contribute to the shock-wave reverberation.

During the shock release, the pressure drops faster than temperature and the coesite single-crystal begin to break and expand, and subsequently melt (decompression melting). At this point, the coesite single-crystal disintegrate in fragments of single crystals, which progressively detach, misalign and melt in the amorphous silica matrix (panel 3 of Fig. 4; Fig. 1E up). This process extends throughout the decompression process as long as pressure reaches ambient condition and post-shock temperature results in the formation of the coesite aggregates up to the almost complete melting of coesite. Here, the coesite aggregates expand and merge each other forming the aggregates of rounded and corroded coesite grains (of about 50 nm) embedded in silica glass (panel 4 of Fig. 4; Fig. 1E down). So, we infer that the silica glass present in the shocked sandstone from Kamil crater originated by the melting of the previously formed coesite and surrounding quartz during the decompression stage. Curiously, only the larger coesite domains show evidence of silica glass, and we observe coesite at different evolutionary stages in an area of few square microns. This is likely connected with the not equilibrated nature of the rock and the spatial and temporal randomisation of coesite nucleation, conceivably connected with the occurrence of local structural features (impurities, fractures or crystal defects) and their interplay with shock wave reverberation.

For both Australasian tektite strewn field and Kamil crater we can assume a peak shock pressure of at least 15 GPa as provided by the presence of PDFs and the amount of silica glass plus HP silica polymorphs^10,11^. Nevertheless, this pressure may have varied significantly throughout the rock, even at the microscopic scale, due to all those interfaces that affect the propagation of the shock wave. Pressure and temperature result therefore heterogeneously distributed in the whole rock. The fact that not all the quartz transformed into coesite, and that only a small fraction of coesite possibly transformed to stishovite^12^, is the consequence of the short time that occurred before pressure dropped to ambient pressure and the different PTt gradients (t stands for time) characteristics of any polymineralic rock. The resulting rock resemble a non-completed reaction and consists then in a mixture of not equilibrated phases, where quartz exists together with coesite, and possibly other high-pressure phases like stishovite^10^. We postulate that the length of time the pressure remains above quartz–coesite border has a major impact on the final quartz-to-coesite volume ratio, rather than the mere value of the peak pressure associated with the shock event. Time may indeed represent the main factor that separates natural shock events from shock experiments. The synthesis of coesite in shock recovery experiments has so far failed, probably due to the too short pressure pulses that can be reached in laboratory (few microseconds), compared to the several milliseconds provided by natural impact scenario^2,20,21^.

We document the formation of coesite through direct quartz > coesite transformation in a very small (Kamil crater) and a very large (Australasian strewn field) impact event. Such a mechanism is thus independent form the size of the impact. Our findings also emphasize the role of shock reverberation at media discontinuities in generating, at least locally, the PTt conditions for the nucleation of coesite through direct transformation from quartz, as previously suggested by Folco et al.^10^ and Campanale et al.^11^. Discontinuities, including primary discontinuities like porosity and grain boundaries, or shock induced discontinuities, like fractures, dislocations, PDFs, nano-defects, etc., occur in all target rocks. This implies that the mechanism proposed here can apply to all quartzose target rocks, regardless of their porous or crystalline nature. In this regard, we point out that what featured in panel 4 of Fig. 4 (which corresponds to the textural setting seen in Fig. 1E down) strongly resemble the typical occurrence of impact-coesite in crystalline rocks. See for instance Fig. 7 of Langenhorst^4^, in which coesite aggregates of 50–100 nm are embedded in amorphous silica from suevite (Ries crater, Germany). Note also the similarity between the coesite textural setting featured in Fig. 1A,B of the present work and the coesite aggregates reported by Jaret et al.^7^ (their Fig. 2E,F) and Ferrière et al.^22^ (their Fig. 8.9) from the Lonar (India) and Bosumtwi (Ghana) impact craters, respectively. Unlike our samples, the latter are fully embedded in diaplectic or silica glass, yet this difference may simply have resulted from a higher degree of melting during the post-shock decompression evolution.

## Methods

### Samples and general procedure

The samples studied in this work are from two different impact sites, namely a thin section of the shocked porous sandstone (reference code L23) from Kamil crater (southern Egypt) and two coesite-bearing quartz ejecta (reference codes SO95A66 and 1144A456) from the Australasian tektite/microtektite strewn field (ODP site 1144A and Sonne Core SO95-17957-2).

All samples were first petrographically investigated using field emission gun – scanning electron microscopy (FEG-SEM). Afterwards, we used focused-ion beam (FIB) for extracting 5 electron-transparent lamellae from the Kamil crater shocked sandstone and 3 lamellae from the Australasian coesite-bearing ejecta (1 from SO95A66, 2 from 1144A456). These lamellae were investigated by transmission electron microscopy (TEM) and 3D electron diffraction^23^ (3D ED) for nano-petrographic and crystallographic analyses. FEG-SEM and Raman spectroscopy of the same samples are reported in Folco et al.^10^ and Campanale et al.^11^.

### Scanning electron microscopy and focused ion beam

FEG-SEM backscatter electron (BSE) images were obtained at the Centro per l'Integrazione della Strumentazione dell'Università di Pisa (CISUP) using a FEI Quanta 450. The 5 electron-transparent lamellae were prepared at the Kelvin Nanocharacterisation Centre of the University of Glasgow using a dual beam FIB FEI 200TEM FIB, following the procedure described in Lee et al.^24^.

### Transmission electron microscopy and electron diffraction

TEM and electron diffraction (ED) studies were carried out at the Center for Nanotechnology Innovation@NEST of the Istituto Italiano di Tecnologia using a ZEISS Libra operating at 120 kV and equipped with a LaB_6_ source and a TRS 2 k × 2 k charge-couple device CCD camera. 3D ED data were acquired using an ASI Timepix detector^25^, capable of collecting the arrival of single electrons and deliver patterns that are virtually background-free. 3D ED data sets were obtained rotating the sample along the tilt axis of the TEM goniometer using the procedure described by Mugnaioli and Gemmi^26^. 3D ED acquisitions were performed in angular steps of 1° and for tilt ranges up to 90°. All data were acquired with a parallel beam of ~ 300 nm through the nano-beam electron diffraction (NED) mode using a 10 µm C2 condenser aperture. We further used an extremely mild illumination in order to avoid any alteration or amorphization of the sample during acquisition. Data were taken from seven coesite crystals in Australasian samples and from two coherent single-crystal coesite in Kamil crater samples. We then reconstructed and analysed the data using the *ADT3D* program^27^ and dedicated *Matlab* routines.

## Supplementary Information

Supplementary Information.
