# Supplementary material for: The formation of impact coesite
Source: Sci Rep. 2021 Aug 6;11:16011. doi: 10.1038/s41598-021-95432-6 (PMC8346461; doi:10.1038/s41598-021-95432-6)

The formation of impact coesite

F. Campanale[1,2], E. Mugnaioli[2], M. Gemmi[2], and L. Folco[1,3]

[1]Dipartimento di Scienze della Terra, Università d Pisa, Via S. Maria 53, 56126 Pisa, Italy

[2]Center for Nanotechnology Innovation@NEST, Istituto Italiano di Tecnologia (IIT), Piazza San Silvestro 12, 56127 Pisa, Italy

[3]Centro per l'Integrazione della Strumentazione dell'Università di Pisa (CISUP), Lungarno Pacinotti 43,

56126 Pisa, Italy

Fig. SM1. FEG-SEM Kamil
A: back scatter electron (BSE) image showing the area of study of the shocked sandstone from Kamil crater, Egypt. The five electron-transparent lamellae extracted with FIB are shown with a yellow rectangle. B: Close-up view of the rectangular area in (A). Panels 1A and 1B match the corresponding panels in Figure 1 of the main text.

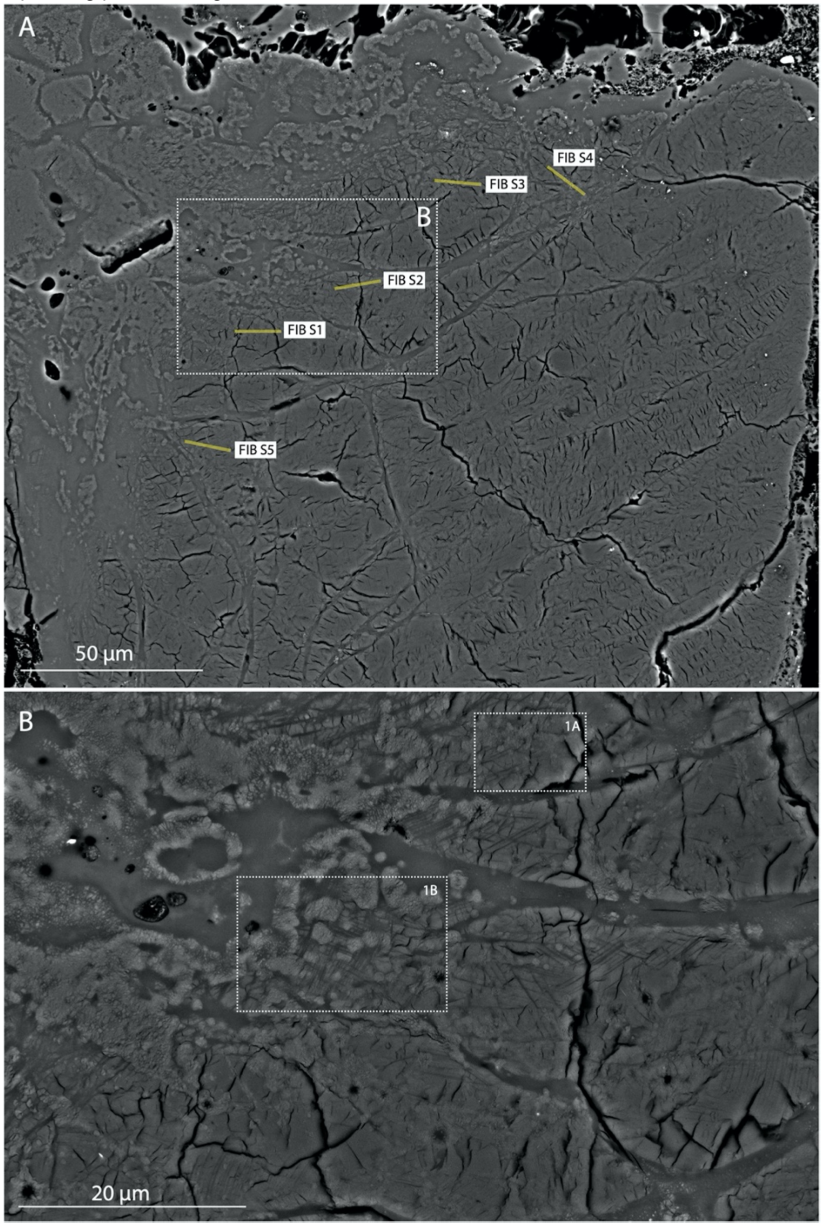

Fig. SM2. FEG-SEM Australasian

BSE images showing the two silica ejecta particles from the Australasian tektite/microtektite strewn field studied in this work. These particles mostly consist of a mixture of coesite and quartz in variable proportion, with up to three sets of planar deformation features (PDFs) in quartz and possibly trace amounts of amorphous silica phases. The yellow rectangles represent the positions of the FIB-lamellae extracted for the TEM investigation.

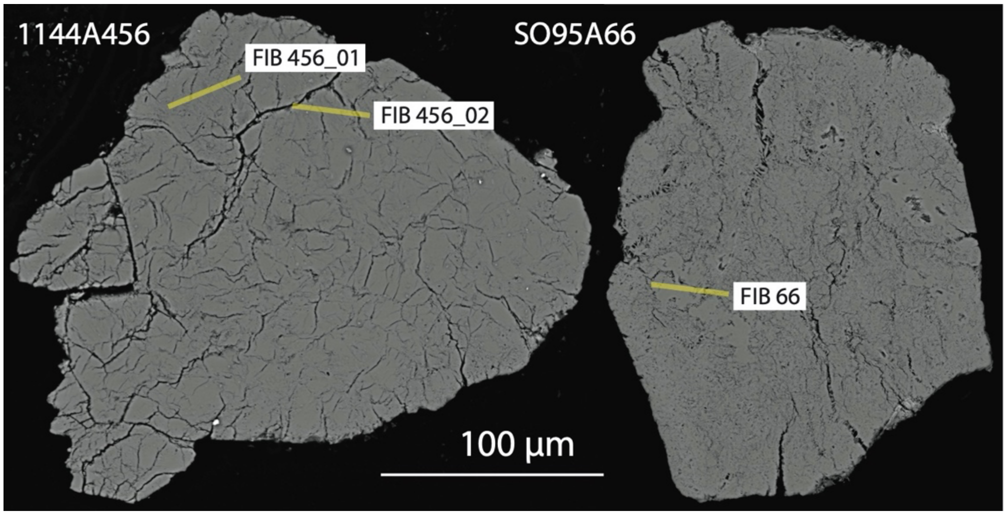

Fig. SM3. TEM Kamil

Bright-field TEM images providing an overview of the five FIB lamellae extracted from the regions indicated in Fig. SM1A, in the shocked sandstone from Kamil crater, Egypt. Panels 1C,D,E match the corresponding panels in Figure 1 of the main text.

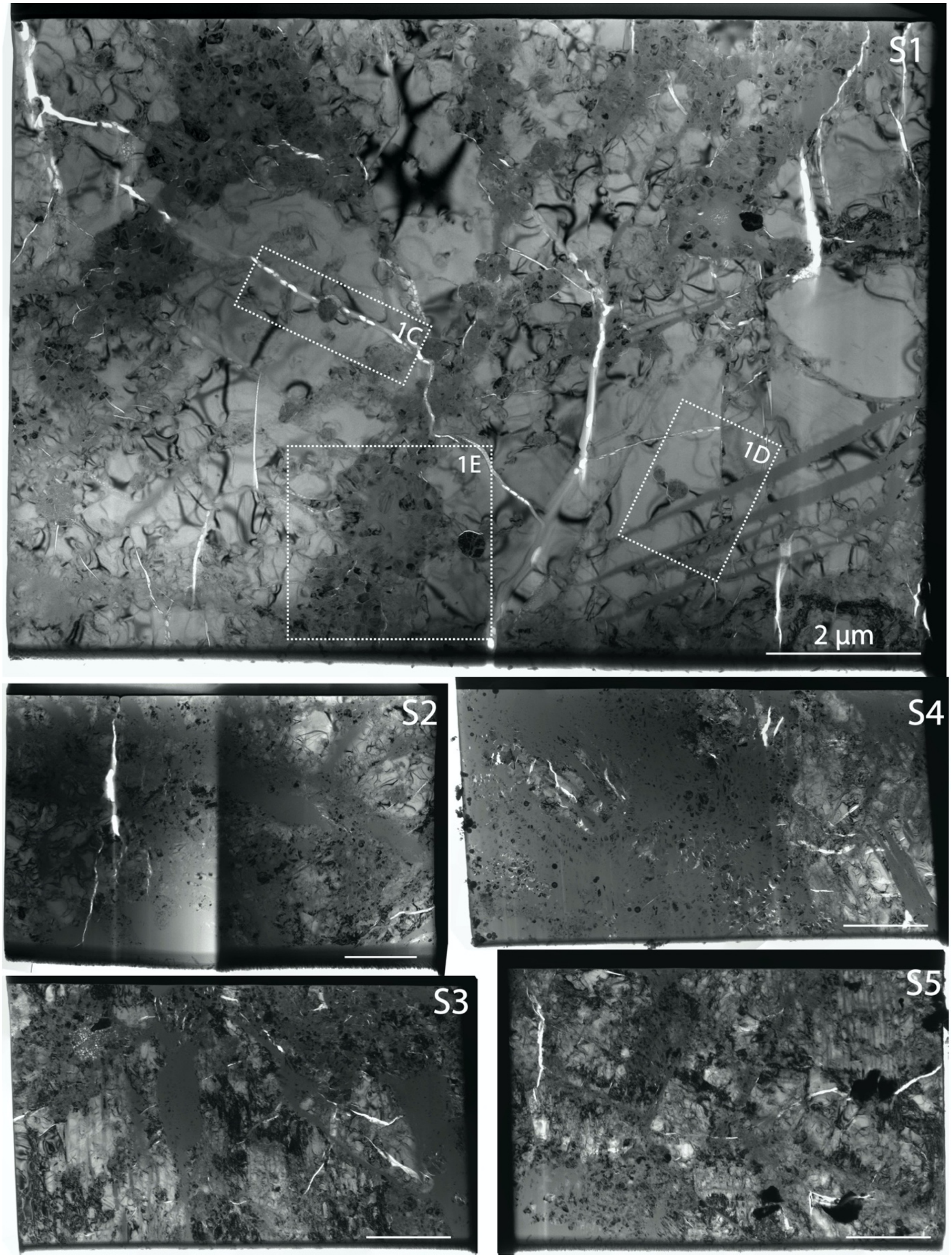

Fig. SM4. TEM Australasian

Bright-field TEM images providing an overview of the three FIB lamellae extracted from the regions indicated in Fig. SM2, in the shocked silica ejecta particles from the Australasian tektite/microtektite strewn field. Panels 2A,B,C,D match the corresponding panels in Figure 2 of the main text.

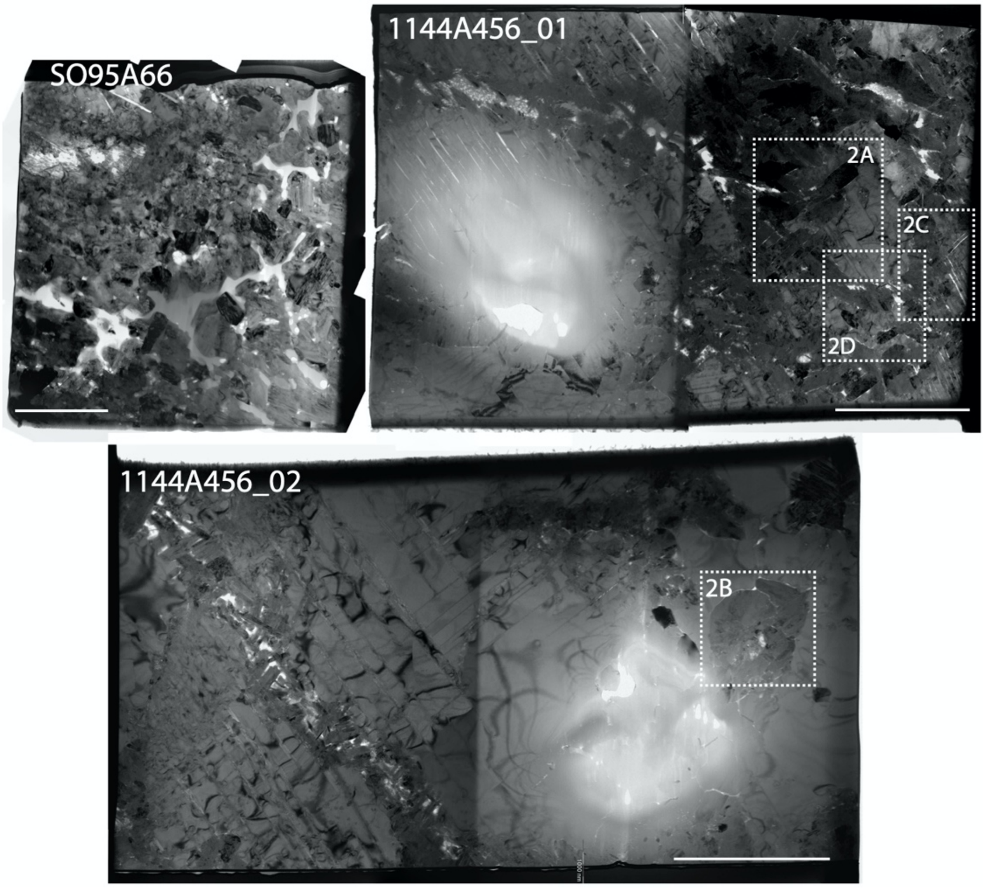

Supplement: Supplementary file 1 — Supplementary Information. [file 41598_2021_95432_MOESM1_ESM.pdf]
